# Social Determinants and Mental Health in Newly Arrived Young Migrants in Spain

**DOI:** 10.1192/j.eurpsy.2024.368

**Published:** 2024-08-27

**Authors:** M. Franch-Roca, A. Gabarrell-Pascuet, E. Salomon-Mallat, M. Group, J. M. Haro, P. Cristóbal-Narváez

**Affiliations:** ^1^Research and Development Unit, Institut de Recerca Sant Joan de Déu, Barcelona; ^2^Centre for Biomedical Research on Mental Health (CIBERSAM), Madrid, Spain

## Abstract

**Introduction:**

Research has shown that factors related with the migratory process (such as travelling alone, living away from family, and discrimination after arrival) considerably increase the risk of mental health problems in young migrants. Moreover, they are among the most vulnerable migration groups with a high risk of social exclusion.

**Objectives:**

To identify coping strategies and behavioural changes used to deal with perceived discrimination and its impact on the emotional well-being and mental health of newly arrived young migrants in Spain.

**Methods:**

A subsample of 15 audio-recorded in-depth qualitative interviews were analysed from the national action-research Migrasalud project (II IN 190517 EN 162 FA 01). The interviews were transcribed, translated from Arabic to Spanish, and analysed through content analysis.

**Results:**

Most participants were males (93.3%; n=14), ranging from 18 to 20 years, and from Morocco (93.3%; n=14). All participants were from foster care placements in Barcelona and arrived to Spain as minors. Newly arrived young migrants reported that they perceived themselves as being healthy before the migratory process. Adverse experiences during the journey and discrimination after arrival impacted their well-being and mental health. Specifically, they reported perceived discrimination in their daily life due to culture, language, or origin. This negatively impacted their well-being and mental health, increasing their emotional distress response and ‘undervalued or inferior’ and ‘vulnerable’ feelings about themselves. Concerning coping with discrimination, they reported using internalised coping strategies such as ‘ignoring’ or ‘not responding’ for fear of having their legal documents revoked or not obtaining them. Their behavioural changes often occurred when they perceived unfair treatment or prejudice towards their migrant status or their socioeconomics, culture or religion. These changes were motivated by being more accepted by the local community by ‘westernisation or cultural assimilation’ and by ‘creating a good image’ of oneself and its culture.

**Conclusions:**

Findings establish that the cumulative experience of post-migration stressors (such as discrimination) negatively impacts their mental health and well-being in the long term. This suggests the need for specific policies and services to address this population’s effects of post-migration risk factors. Further research is needed to explore the causes and effects of perceived discrimination on mental health more closely and to develop more targeted and effective interventions.

**Disclosure of Interest:**

None Declared

